# Community-acquired pneumonia in the United Kingdom: a call to action

**DOI:** 10.1186/s41479-017-0039-9

**Published:** 2017-10-05

**Authors:** James Chalmers, James Campling, Gillian Ellsbury, Peter M. Hawkey, Harish Madhava, Mary Slack

**Affiliations:** 1 University of Dundee, Ninewells Hospital and Medical School, Dundee, DD1 9SY UK; 2 Pfizer Ltd, Tadworth, KT20 7NS UK; 3 Institute of Microbiology and Infection, University of Birmingham, B15 2TT, Birmingham, UK; 4 School of Medicine, Griffith University, Campus, Gold Coast, QLD 4222 Australia; 5 Division of Molecular & Clinical Medicine, School of Medicine, Ninewells Hospital and Medical School, Dundee, DD1 9SY UK

**Keywords:** Antimicrobial resistance, *Clostridium Difficile*, Community-acquired pneumonia, Immunization, Pneumococcal disease, Pneumonia burden, Pneumonia diagnostics, Pneumonia epidemiology, *Streptococcus Pneumoniae*

## Abstract

Pneumococcal disease has a high burden in adults in the United Kingdom (UK); however, the total burden is underestimated, principally because most cases of community-acquired pneumonia (CAP) are non-invasive. Research into pneumonia receives poor funding relative to its disease burden (global mortality, disability-adjusted life years, and years lived with disability), ranking just 20 out of 25 for investment in infectious diseases in the UK. The current accuracy of data for establishing incidence rates is questionable, and it is a reflection of the paucity of research that much of the background information available derives from nearly 30 years ago. Given the relationship between CAP and mortality (pneumonia accounts for 29,000 deaths per annum in the UK, and 5–15% of patients hospitalised with CAP die within 30 days of admission), and the increasing threat of antimicrobial resistance associated with inappropriate antibiotic prescribing, such neglect of a highly prevalent problem is concerning. In this Call to Action, we explore the poorly understood burden of CAP in the UK, discuss the importance of an accurate diagnosis and appropriate treatment, and suggest how national collaboration could improve the management of an often life-threatening, yet potentially preventable disease.

## Background

By any measure, pneumonia has a huge impact on the United Kingdom (UK) and European healthcare systems, being associated with high rates of hospital admission and length of stay. Across Europe, annual inpatient care accounts for healthcare expenditure of €5.7 billion, outpatient care for €0.5 billion, and medication for €0.2 billion. The reported incidence of invasive pneumococcal disease (IPD) in the UK is 6.85 per 100,000 annually [1]. In addition, 5–15% of patients hospitalised with community-acquired pneumonia (CAP) will die within 30 days of admission, rising to 30% for those admitted to the intensive care unit [2]. This is particularly worrying because pneumonia is responsible for more hospital admissions and bed days than any other lung disease in the UK, and results in 29,000 deaths per annum—the third greatest cause of death from lung disease after chronic obstructive pulmonary disease (COPD; second greatest cause) and lung cancer (leading cause). Furthermore, the UK ranks 21 out of 99 countries for age-standardized mortality due to pneumonia [3]. CAP also has long-term implications for subsequent mortality; 1-, 5-, and 7-year mortality rates in patients who recovered from CAP in the Netherlands were significantly higher at 17%, 43%, and 53%, respectively, than the mortality rates seen in age- and sex-matched population controls (4%, 19%, and 24%). Malignancy (27%), COPD (19%), and cardiovascular disease (16%) were the most common causes of death [4].

Conditions such as cardiovascular disease have seen mortality rates drop significantly over the past 10 years [5] in line with major research initiatives and funding allocation, but little progress has been observed in pneumonia epidemiology, pathophysiology, or therapy. Indeed, in an analysis of UK infectious disease research funding (1997–2013), pneumonia received poor investment relative to its disease burden (global mortality, disability-adjusted life years, and years lived with disability), ranking just 20 out of 25 infectious diseases [6]. In this article, we argue that, despite its obvious impact and burden, pneumonia is a substantially underestimated, neglected, and underfunded condition in the UK. Many possible reasons exist for this unfortunate position; none of them, we would argue, is acceptable.

*Streptococcus pneumoniae* is the leading cause of community-acquired pneumonia in the UK and Europe [7]. The results of a recent systematic review [2] show that (i) vaccine-type pneumococcal disease still has a high burden in UK adults, and (ii) the total burden of pneumococcal disease in the UK is underestimated, principally because most cases of CAP are non-invasive. Given the relationship between CAP and mortality, and the increasing threat of antimicrobial resistance (AMR) associated with inappropriate antibiotic prescribing, this neglect of a highly prevalent problem is concerning.

Here, we explore the poorly understood burden of CAP in the UK, discuss the importance of an accurate diagnosis and appropriate treatment, and suggest how national collaboration could improve the management of an often life-threatening, yet potentially preventable disease.

## Community-acquired pneumonia is an immediate and growing concern

Pneumonia disproportionately affects older people [8], with an overall CAP incidence of approximately 7.99/1000 person-years in patients aged 65 years or older, and a doubling of incidence between individuals aged 65–69 and 85–89 years, according to 1997–2011 data from the UK Clinical Practice Research Datalink, associated with the Hospital Episode Statistics (HES) database [9]. Given that the UK population is aging (it is estimated that 23% will be aged ≥65 years by 2035 vs. 17% in 2010) [10], the economic burden of caring for elderly patients with pneumonia can only increase in the absence of steps to minimize the incidence of the disease [7].

Pneumonia and lower respiratory tract infections are major causes of morbidity and mortality among those aged 65 years or older [9], and CAP in the elderly can aggravate underlying comorbidities (e.g. cardiovascular disease, renal disease, liver disease, and malignancy) with serious consequences [11]. Furthermore, long-term quality of life is substantially affected by CAP, and pneumococcal pneumonia increases the risk of pneumonia-related mortality three-fold versus non-pneumococcal pneumonia in elderly patients [7].

Given the growing burden of disease, mechanisms to reduce societal- and healthcare-associated costs must be a priority. Prevention aside, the identification of individuals who could be managed in the outpatient setting could not only virtually eliminate hospital costs but also decrease risk of infection with potentially resistant nosocomial bacteria [7]. However, a study by Woodhead et al. [12] (1987), conducted almost 30 years ago, was the last to investigate the relative proportions of patients with CAP accessing primary and secondary care in the UK. This study found that 22% of CAP was treated in hospital, with the remainder treated in primary care [12]. For the UK National Health Service (NHS), avoiding emergency admissions is a major concern due to the high costs versus other forms of care; however, most clinical commissioning groups (formerly known as primary care trusts) still have high rates of emergency admissions [13].

Increased socioeconomic deprivation is associated with increased incidences of both CAP and lower respiratory tract infection. Regional variations exist; rates of CAP in the UK are approximately 70% higher in the most deprived quintile (North England) than in the least deprived quintile (London and South East coast) [9].

Finally, CAP has an indirect socioeconomic impact; the same historical cohort study [12] mentioned above found that approximately half of patients in employment required more than two weeks off work. Data are lacking on the current effect of pneumonia on work days lost to CAP in the UK. In Europe, this cost is estimated to be €3.6 billion annually [7].

## How are policy makers and healthcare funders making informed decisions?

**Fig. 1:**
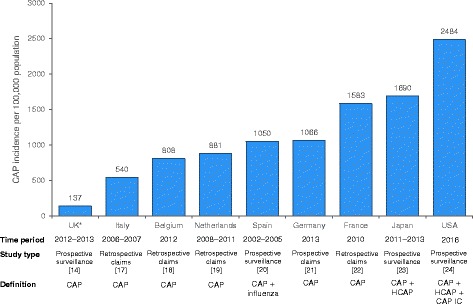
Incidence rates of CAP around the world. *Age group 65–74 years only. CAP, community-acquired pneumonia; CAP IC, community-acquired pneumonia in children; HCAP, healthcare-acquired pneumonia

CAP is a cause for serious concern, yet it is largely ignored in the political and healthcare arenas. This situation could reflect a lack of understanding of the extent of the problem. The current accuracy of data for establishing incidence rates is questionable; we believe the statistics quoted above are an underestimate. This view is supported by data from other developed countries (see Fig. 1). It is a reflection of the paucity of research that much of the UK background information available derives from nearly 30 years ago.

In more detail, the recent prospective cohort study of adults (aged ≥16 years) with CAP admitted to two large teaching hospitals (acute admission units, hospital wards and critical care units) in Nottingham, UK, used a standardized proforma to collect daily information on patient demographics, clinical information, microbiological investigations, radiological findings and outcome measures [14]. Inclusion criteria comprised symptoms suggestive of lower respiratory tract infection (at least one of breathlessness, cough, sputum, or fever), with new infiltrates on chest radiography consistent with pneumonia, and treatment by the admitting clinical team for CAP. Exclusion criteria were post-obstruction pneumonia due to lung cancer, active tuberculosis (discharged from hospital within the preceding 10 days), and aspiration pneumonia. The overall incidence rates for patients who were hospitalized with CAP and pneumococcal CAP over 5 years (2008–2013) were 79.9 and 23.4 cases per 100,000 population, respectively. However, this study was not strictly designed to determine the overall incidence of CAP. It did not, for example, include data for patients (i) who did not consent for study; (ii) from whom a urine sample was not obtained; (iii) who were discharged from hospital within 10 days previously; (iv) who were admitted via a route not involving acute admission units, hospital wards and critical care units, or (v) who attended Accident and Emergency (A&E) but were not admitted. Furthermore, a substantial discrepancy exists between the number of patients aged 16 years or older and eligible for inclusion in the Nottingham study (*n* = 2702) [14] and HES data for the corresponding population (*n* = 11,059) [15]; there is also a large difference in CAP incidence rates from these two sources. Miscoding of HES data is a well-recognized limitation of the database, but that notwithstanding, the four-fold scale of this range is alarming, not least because it is unclear which of the two values is more accurate. Such a discrepancy might represent the difference between, for example, an incidence in Nottingham of 50,000 and 200,000 CAP cases per annum. These data together with known, extensive regional variations associated with socioeconomic deprivation [9] and higher European incidence rates (Fig. 1) would tend to reduce confidence in published UK incidence rates. HES data are used by Public Health England (PHE) to help guide policy [16], but the problems outlined above mean that decisions are based on data which might lack adequate strength and/or consistency.

**Fig. 2:**
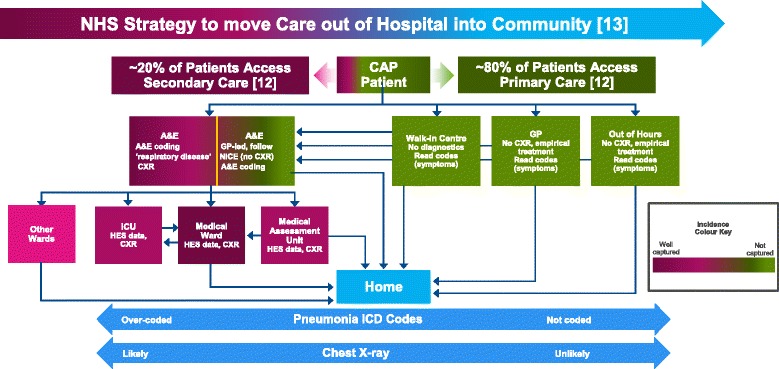
Simplified schematic of the UK care pathway for adult patients with CAP accessing healthcare, and estimated capture of incidence rates. A&E, accident and emergency; CAP, community-acquired pneumonia; CXR, chest X-ray; GP, general practitioner; HES, hospital episode statistics; ICU, intensive care unit; NICE, National Institute for Health and Care Excellence

A comparison of rates across Europe suggests that the incidence of CAP is seriously underestimated in the UK (Fig. 1), lending credence to the suggestion that the Nottingham study data may represent an incomplete assessment of the incidence of CAP in the UK, which the HES data might help to clarify. The authors considered opinion is that even the latter numbers are likely to be an underestimate. By considering possible routes via which patients with CAP access healthcare in the UK (Fig. 2), it becomes clear that across the country, the capture of CAP incidence in primary care is difficult, and the capture of CAP incidence in secondary care is incomplete. One of the fundamental drivers of this problem is the lack of a specific ICD-10 (10th revision of the International Statistical Classification of Diseases and Related Health Problems) diagnostic code for CAP, with the result that coding is not complete and patients are spread across multiple diagnostic codes.

The consequence of underestimating the incidence, and therefore the importance, of CAP is that its impact on major healthcare outcomes such as AMR, *Clostridium difficile* infection (CDI), healthcare costs and winter pressures are, in turn, greatly underestimated. Such incomplete data on the incidence of CAP have major implications for all involved in healthcare, but particularly for those responsible for healthcare policy at governmental and local levels, who base far-reaching decisions on such information. It is our view that we cannot tackle the consequences of CAP until we raise the profile of CAP among the public, policy makers, and research funders.

## Inappropriate antibiotic treatment of community-acquired pneumonia aggravates the development of antimicrobial resistance

Hospitalization for CAP is increasing; from 1998 to 2008, the incidence of CAP-associated admissions in Oxfordshire (UK) rose by 4.2% per year, accelerating to 8.8% per year from 2009 to 2014 [8]. Trotter et al. [17] also showed a marked increase (34%) in pneumonia hospitalizations between 1997 and 1998 and 2004–2005 in the UK. Consequently, because antibiotics are most commonly indicated for respiratory tract infections in UK hospitals (comprising 31% of prescriptions) [18], it seems likely that increasing rates of hospital admissions for CAP will also result in a rise in antibiotic prescribing, contributing to the development of AMR [8].

In Europe, AMR has been observed in all pathogens associated with CAP, including *S. pneumoniae*, which is the single most common causative agent isolated [7]. CDI is strongly associated with broad-spectrum antibiotic use in CAP and is often nosocomial. [19, 20] A study [20] in two Edinburgh hospitals found that (i) all of the broad-spectrum antibiotics commonly used in CAP (amoxicillin/clavulanic acid, cephalosporins, and quinolones) were associated with a high level of risk for CDI, and (ii) shortened antibiotic treatment duration can reduce disease incidence, risk of developing AMR, side effects, length of stay and hospital costs.

Improved antimicrobial stewardship and the development of novel measures to tackle AMR are urgently needed. A pathogen-directed antibiotic strategy (e.g. use of penicillin rather than amoxicillin-clavulanate to treat likely or confirmed pneumococcal disease) has demonstrated comparable clinical efficacy to an empirical broad-spectrum antibiotic strategy in patients with CAP [21]. Appropriate treatment with pathogen-directed antibiotics is likely to help reduce the risk of AMR, but we lack robust, cost-effective and widely available diagnostics. A perception exists that antimicrobial-resistant pathogens are increasing in UK and international CAP patients, leading to increased use of broad-spectrum antibiotics. Without prospective studies using modern diagnostics and determining the true incidence of CAP and its associated pathogens, antibiotic policies are reliant on superannuated microbiological data, or international data that may not be applicable to the UK. A systematic review has shown that current criteria used to identify potentially antibiotic resistant pathogens in the USA are not applicable to UK or European CAP patients [22].

There is an urgent need to develop rapid, accurate, point-of-care diagnostics capable of (i) differentiating between viral and bacterial infections in CAP in the community setting to minimize unnecessary antibiotic prescription [23], and (ii) identifying bacterial infections to guide pathogen-directed antibiotic treatment. Point-of-care diagnostics will have added benefit in helping to establish both the true incidence of CAP and the understanding of bacterial versus viral burden in the disease. Initiatives such as the “Longitude Prize” [24] are an important factor in promoting diagnostic research.

A 2013 Cochrane review [25] has shown that 23-valent pneumococcal polysaccharide vaccination prevents IPD and non-invasive pneumococcal pneumonia, but does not have an impact on all-cause CAP. It is essential to increase vaccine coverage (e.g. against the most common causative agents, *S. pneumoniae* and influenza) [7]) to at least prevent IPD and non-invasive pneumonia, thereby potentially reducing antibiotic use (including that for treating pneumonia-associated secondary infection) and minimizing selective pressure leading to AMR [23]. The WHO Global Action Plan on Antimicrobial Resistance advocates the development and use of new or improved vaccines to prevent diseases becoming problematic due to AMR [26]. Furthermore, the UK Joint Committee on Vaccinations and Immunisation (JCVI) has recognized the strategic importance of immunization in addressing AMR, recommending that cost-effectiveness analyses of vaccination programs should include the potential benefits of reduced antimicrobial use [27].

Data from the Nottingham study [14], together with serotype-specific surveillance data for IPD (July to June from 2002 to 2003 to 2013–2014) collated by Public Health England, published by Waight et al. [1] and analyzed for cost effectiveness by Van Hoek et al. [28], were instrumental in the JCVI decision that 13-valent pneumococcal conjugate vaccine would not be universally recommended for those aged 65 years and older in England. The vaccine is therefore only offered to those aged 10 years or older who have been identified as being at particularly high risk of, and high mortality from, IPD (e.g. those receiving bone marrow transplants, or with acute or chronic leukaemia) [29]. Necessarily, determining the efficacy and cost effectiveness of vaccination programs requires accurate information on the burden of disease. As noted previously, data on CAP incidence are poorly captured in the UK, not least because most cases of CAP are non-invasive [2].

## Call to action

As a first step, we draw attention to pneumonia as an underestimated, neglected, and underfunded condition in the UK, and call for all healthcare practitioners, researchers, health planners, and policy makers at both primary and secondary care level to react swiftly, as outlined in Table 1. National prospective studies of the true incidence of CAP in both primary and secondary care (including reasons for patients being overlooked or lost to follow-up), immediate review of CAP diagnostic methods, and effective preventative strategies (e.g. adequate vaccination programs) are urgently needed to ensure that all patients (especially the elderly) receive optimal care and treatment to minimize the impact of CAP, reduce its medical and socioeconomic burden, and restrict the development of AMR.

**Table 1:** Call to Action in the UK

Implement core research into CAP incidence and development of diagnostic tests	• Prospective, national community study of the true incidence of CAP in primary care o Incorporating representative centres from each major UK geographical region • Prospective national study of the true incidence of CAP in secondary care o Involving ~5 representative centres; one in each major UK geographical region • Develop simple, accurate, and affordable point-of-care diagnostic(s) for: o Differentiating viral vs. bacterial CAP to avoid unnecessary antibiotic use o Identifying causative pathogen to guide pathogen-directed antibiotic therapy
Investigate the true impact of CAP on AMR	• Role/scale of inappropriate antibiotic prescribing in the treatment of CAP in both primary and secondary care • Contribution of inappropriate prescribing in CAP to the development of AMR • Establish the effectiveness of antibiotic stewardship programs in terms of improved CAP outcomes and reduced AMR • Comprehensive molecular diagnostic studies in patients with CAP to establish the incidence of antibiotic resistant pathogens and novel approaches to identify those requiring broad-spectrum antibiotic therapies
Implement appropriate immunization	• Institute appropriate vaccination strategy, including groups at risk, according to national recommendations

## Abbreviations

A&E: accident & emergency
AMR: antimicrobial resistance
CAP IC: community-acquired pneumonia in children
CAP: community acquired pneumonia
CDI: *Clostridium difficile* infection
CXR: chest X-ray
GP: general practitioner
HCAP: healthcare-acquired pneumonia
HES: Hospital Episode Statistics
ICD-10: International Statistical Classification of Diseases and Related Health Problems – 10th revision
ICU: intensive care unit
IPD: invasive pneumococcal disease
JCVI: Joint Committee on Vaccinations and Immunisation
NHS: National Health Service
NICE: National Institute for Health and Care Excellence
PHE: Public Health England
WHO: World Health Organization
